# The Global Research of Artificial Intelligence on Prostate Cancer: A 22-Year Bibliometric Analysis

**DOI:** 10.3389/fonc.2022.843735

**Published:** 2022-03-01

**Authors:** Zefeng Shen, Haiyang Wu, Zeshi Chen, Jintao Hu, Jiexin Pan, Jianqiu Kong, Tianxin Lin

**Affiliations:** ^1^ Department of Urology, Sun Yat-Sen Memorial Hospital, Sun Yat-sen University, Guangzhou, China; ^2^ Graduate School, Tianjin Medical University, Tianjin, China; ^3^ Guangdong Provincial Key Laboratory of Malignant Tumor Epigenetics and Gene Regulation, Guangzhou, China

**Keywords:** artificial intelligence, prostate cancer, bibliometric, VOSviewer, Citespace

## Abstract

**Background:**

With the rapid development of technology, artificial intelligence (AI) has been widely used in the diagnosis and prognosis prediction of a variety of diseases, including prostate cancer. Facts have proved that AI has broad prospects in the accurate diagnosis and treatment of prostate cancer.

**Objective:**

This study mainly summarizes the research on the application of artificial intelligence in the field of prostate cancer through bibliometric analysis and explores possible future research hotspots.

**Methods:**

The articles and reviews regarding application of AI in prostate cancer between 1999 and 2020 were selected from Web of Science Core Collection on August 23, 2021. Microsoft Excel 2019 and GraphPad Prism 8 were applied to analyze the targeted variables. VOSviewer (version 1.6.16), Citespace (version 5.8.R2), and a widely used online bibliometric platform were used to conduct co-authorship, co-citation, and co-occurrence analysis of countries, institutions, authors, references, and keywords in this field.

**Results:**

A total of 2,749 articles were selected in this study. AI-related research on prostate cancer increased exponentially in recent years, of which the USA was the most productive country with 1,342 publications, and had close cooperation with many countries. The most productive institution and researcher were the Henry Ford Health System and Tewari. However, the cooperation among most institutions or researchers was not close even if the high research outputs. The result of keyword analysis could divide all studies into three clusters: “Diagnosis and Prediction AI-related study”, “Non-surgery AI-related study”, and “Surgery AI-related study”. Meanwhile, the current research hotspots were “deep learning” and “multiparametric MRI”.

**Conclusions:**

Artificial intelligence has broad application prospects in prostate cancer, and a growing number of scholars are devoted to AI-related research on prostate cancer. Meanwhile, the cooperation among various countries and institutions needs to be strengthened in the future. It can be projected that noninvasive diagnosis and accurate minimally invasive treatment through deep learning technology will still be the research focus in the next few years.

## Introduction

Artificial intelligence (AI) is a subject that mainly studies the application of computers to simulate human intelligent behavior, involving computing, mathematics, biology, and many other subjects (1). With the updating and progress of machine learning (ML) and deep learning (DL), AI has been applied to many fields, especially in the fields of image recognition, intelligent control, and program automation design (2, 3). AI has been applied to the auxiliary diagnosis of disease since the 1950s (4). With its powerful algorithms and learning capabilities, AI has gradually been widely used in all aspects of medical and health, including disease diagnosis, prognosis prediction, drug research, genomics data analysis and other related fields, bringing new innovations and methods to medical field, which are conducive to the development of precision medicine (5, 6).

According to the GLOBOCAN cancer statistics for 2020 from the International Agency for Research on Cancer (IARC) (7), prostate cancer (PCa) is the third most commonly diagnosed malignant carcinoma in the world and the most common tumor of genitourinary system in males. Therefore, higher diagnostic accuracy and precise treatment are of great significance to the prognosis of patients with PCa. At present, prostate biopsy is the gold standard for the diagnosis of PCa (8). It is an invasive operation and its indications are mainly based on the level of prostate-specific antigen (PSA), prostate MR, and digital rectal examination, which can easily lead to overtreatment or undertreatment (9–12). AI, to a certain extent, can improve the diagnostic accuracy of PCa, achieving precision medicine.

At present, with the continuous development of radiomics, digital pathology, and genomics, AI based on ML and DL has many applications in the diagnosis, tumor staging or grading, treatment, and prognosis of PCa. Based on clinical data, pathological images, and MRI images, many AI technologies such as ML, Artificial Neural Network (ANN), or Deep Learning System (DLS) are used for the diagnosis and Gleason score of PCa, and most studies have achieved good prediction ability (13–17). Furthermore, automatic grading of PCa can be realized through digital pathology by using a hybrid approach of Convolutional Neural Network (CNN) and handcrafted features (18–20). Meanwhile, researchers utilized CNN to achieve precise prostate biopsy or treatment through MRI and transrectal ultrasonography (TRUS)-targeted or fusion biopsy (14, 21). In genomics research, AI has also been used to identify specifically expressed genes with clinical significance, to achieve tumor risk stratification and individualized treatment (22). For the evaluation of treatment and prognosis of PCa, it is reported that scholars take use of clinical data and image data to predict the recovery situation and biochemical recurrence, providing valuable suggestions for clinicians (23–25).

As the interest in the research of AI application in the field of PCa has increased sharply, and a large number of related papers have been published, it is difficult for researchers to clarify the newest developments and research hotspots of this field. According to the current research, AI is still developing rapidly and is still in the preliminary application in the field of PCa. Summarizing its global research trends and research hotspots are of great significance to the next research. Our research team also conducted some AI research in the field of urogenital neoplasms (26–28). However, there is no research on bibliometric analysis to summarize.

Bibliometric analysis (29–33), which has been widely applied in many fields, is an information visualization method to comprehend the knowledge structure and identify the research frontiers or hotspots of a certain field by summarizing all the literature of this field around the world and quantitatively analyzing the literature data and metrological characteristics by using mathematical and statistical methods. Meanwhile, by using this method, we can also compare the research status of various countries, institutions, authors, or journals through the paper information from the database, so as to evaluate global scientific articles and the latest frontier research progress, better understand scientific publications, and visualize their trends (34–36).

Therefore, we determine the countries, institutions, authors, or journals with the highest citations/publications of AI in the field of PCa by collecting literature data in the database. The aim of this study is to summarize the application and development of AI in the PCa from 1999 to 2020 through bibliometric analysis and provide the current research progress, hotspots, and the emerging trends of AI in PCa, which may help new researchers better grasp future research interest.

## Methods

### Database

We used The Science Citation Index Expanded (SCI-Expanded) of Clarivate Analytics’ Web of Science Core Collection (WoSCC) as the data source. The WoSCC is the most frequently used and acceptable database for scientific or bibliometric studies. It contains nearly 9,000 of the world’s most prestigious high-impact journals and more than 12,000 academic conferences.

### Searching Strategy

We searched the information of publications about AI in the field of PCa within 1 day, in order to ensure no data updates. Information regarding titles, keywords, abstracts, authors, and institutions and reference records of the papers were downloaded and saved in plain text format. The searching query string was described as follows: topic=(“artificial intelligence” OR “robotic*” OR “expert* system*” OR “intelligent learning” OR “feature* extraction” OR “feature* mining” OR “feature* learning” OR “machine learning” OR “feature* selection” OR “unsupervised clustering” OR “image* segmentation” OR “supervised learning” OR “semantic segmentation” OR “deep network*” OR “Bayes* network” OR “deep learning” OR “neural network*” OR “neural learning” OR “neural nets model” OR “artificial neural network” OR “data mining” OR “graph mining” OR “data clustering” OR “big data” OR “knowledge graph”) AND topic=(prostate OR prostatic) NEAR/1 (cancer* OR tumor* OR tumour* OR oncology OR neoplasm* OR carcinoma*) AND publication year= (1999–2020). The language was limited to English, and the document types we searched for were also limited to original articles and reviews.

### Data Analysis

The data were downloaded and analyzed by two researchers respectively to assure the accuracy of data and the repeatability of the research. Microsoft Excel 2019 and GraphPad Prism 8 were applied to analyze the targeted files and exported the bar charts and tables of top-cited or productive authors, countries/regions, publications, journals, and institutions. H-index (37), proposed by Hirsch, was a mixed index, which could be used as a significant indicator of appraising both the number and level of academic output of a scientific researcher, country, journal, or institution.

### Data Visualization

In this study, we mainly used VOSviewer (version 1.6.16) (38, 39), Citespace (version 5.8.R2) (29, 40, 41), and a widely used online platform for bibliometric analysis (42) to achieve data visualization. Co-authorship, co-citation, and co-occurrence analysis are the most frequently used measures in bibliometric analysis (43). Co-authorship analysis is to analyze the relationship between authors, countries, or institutions through the number of papers completed jointly. Co-occurrence analysis is a quantitative method to analyze the relationship among different items according to whether the items appear together. Co-citation analysis shows the relationship strength of cited items through the number of citing items (44, 45).

VOSviewer is a widely used software applied for constructing and visualizing bibliometric networks. In this research, VOSviewer was applied to perform the citation/co-citation analysis of country/region and institution and keyword co-occurrence analysis. Meanwhile, we also used Citespace for the co-authorship analysis of institution and author, and co-citation analysis of author, reference and journal. Also, a dual-map overlay of journal was generated by Citespace. Apart from that, an online platform for bibliometric analysis was adopted for country/region co-authorship and publication analyses.

### Research Ethics

The data sources of our study were available from the public databases. Permission from the ethics committee is not needed.

## Results

### Global Trend of Publications and Citations

**Figure 1:**
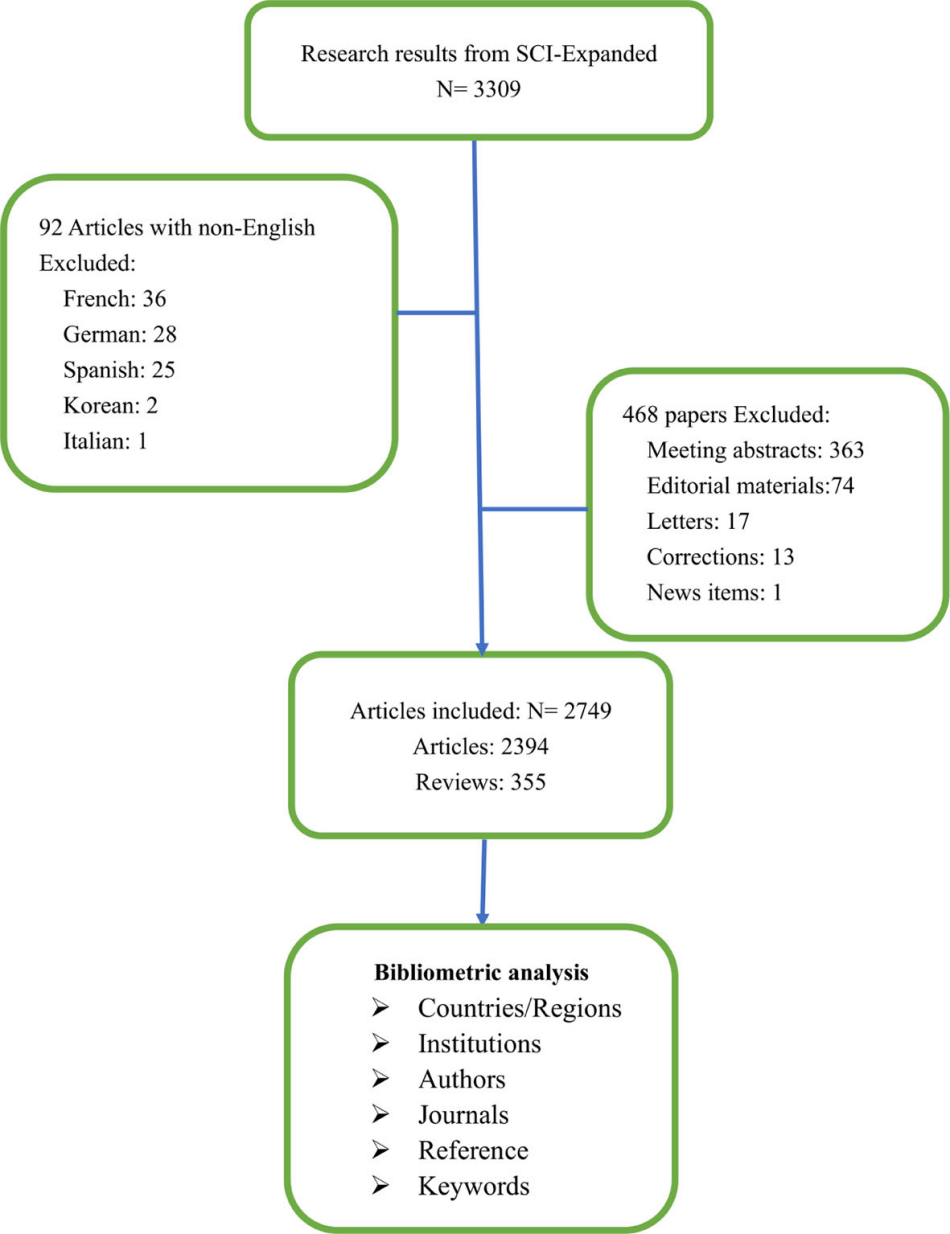
Flowchart of the searching stagey in the study.

**Figure 2:**
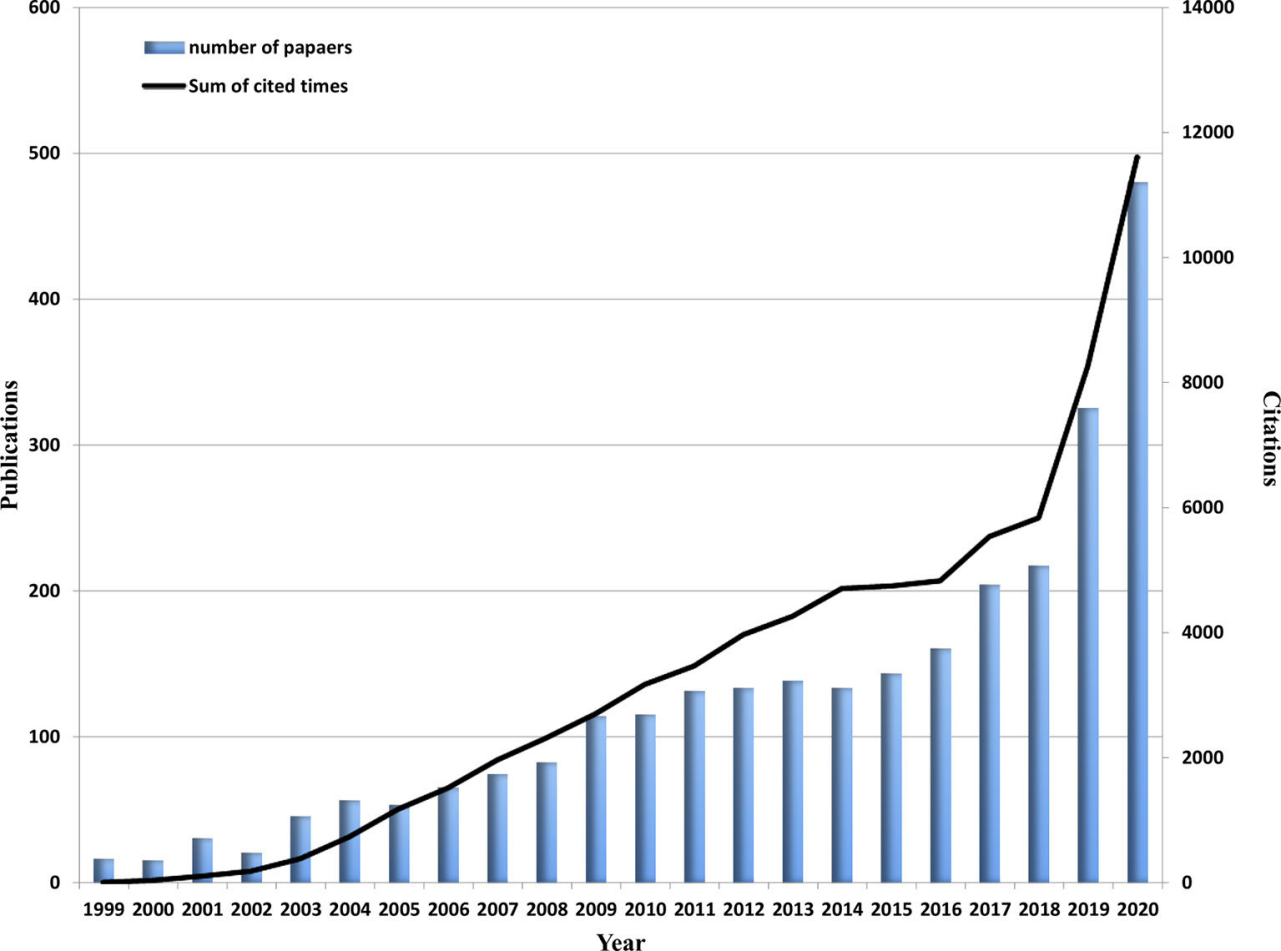
Global trend of publications and total citations on AI research in prostate cancer from 1999 to 2020.

According to the data searching strategy, we collected 2,749 papers including 2,394 original articles and 355 reviews from WoSCC in the last 22 years (**Figure 1**). It could be seen from **Figure 2** that since 1999, the research on AI in PCa has increased every year. Especially in the past 5 years, the research has developed rapidly, accounting for almost 50% of all publications. As of the search date, all papers have been cited 80,373 times, and the H-index and average citations per item are 113 and 29.24. At present, the application of AI in PCa is still the focus of the research.

### Analysis of Top Productive Countries/Regions

**Figure 3:**
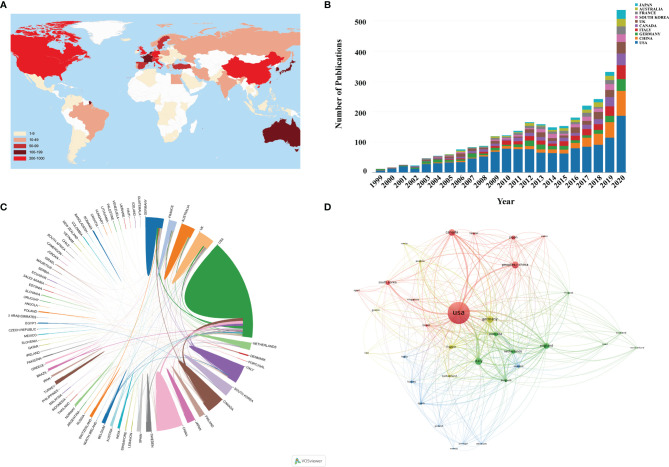
**(A)** World map based on the total publications of different countries/regions. **(B)** The changing trend of the annual publication quantity in the top 10 countries/regions from 1999 to 2020. **(C)** The international collaborations’ visualization map of countries/regions. The thickness of the line between countries reflects the frequency of the cooperation. **(D)** The countries/regions’ citation network visualization map generated by using VOSviewer. The thickness of the lines reflected the citation strength.

A total of 82 countries/regions had published related articles in this field. As can be seen from the world map in **Figure 3A**, countries that had published more than 200 articles include USA, China, UK, Italy, Germany, and Canada. **Figure 3B** shows the annual publication trend of the top 10 countries in recent 22 years. It could be found that the USA ranked first with 1,342 articles from **Table 1**, followed by China (252), Germany (232), and Italy (232). Moreover, the H-index (102) and the total citations (52,685 times) of the USA also ranked first, much higher than that of the second-ranked Germany (47, 10,186 times). When it comes to the collaboration analysis of countries/regions, the USA had the cooperation of many countries, the most important of which were Canada, China, and Italy. However, the cooperation relationship among other countries was weak (**Figure 3C**). We also used the VOSviewer to analyze the cooperation of different countries, and as shown in **Figure 3D**, 34 countries were included when the minimum number of publications was limited in more than 10. The lines between nodes indicated the co-authorship between countries, and the thicker of the line was the stronger of the cooperation [named as total link strength (TLS)]. The co-authorship visualization map showed that the top 5 TLS were the USA, Italy, Germany, Canada, and England.

**Table 1:** Top 10 productive countries/regions related to AI on PCa.

Rank	Country	Counts	Percentage	H-index	Total citations	Average citation per paper
1	USA	1,342	48.818	102	52,685	39.26
2	China	252	9.167	34	7,094	28.15
3	Germany	232	8.439	47	10,186	43.72
4	Italy	232	8.439	45	8,952	38.59
5	Canada	218	7.93	44	7,903	36.25
6	England	198	7.203	42	7,118	35.95
7	South Korea	140	5.093	22	2,147	15.34
8	France	133	4.838	33	4,476	33.65
9	Australia	119	4.329	32	5,127	43.08
10	Japan	118	4.292	25	1,961	16.62

### Contributions of Top Institutions

**Figure 4:**
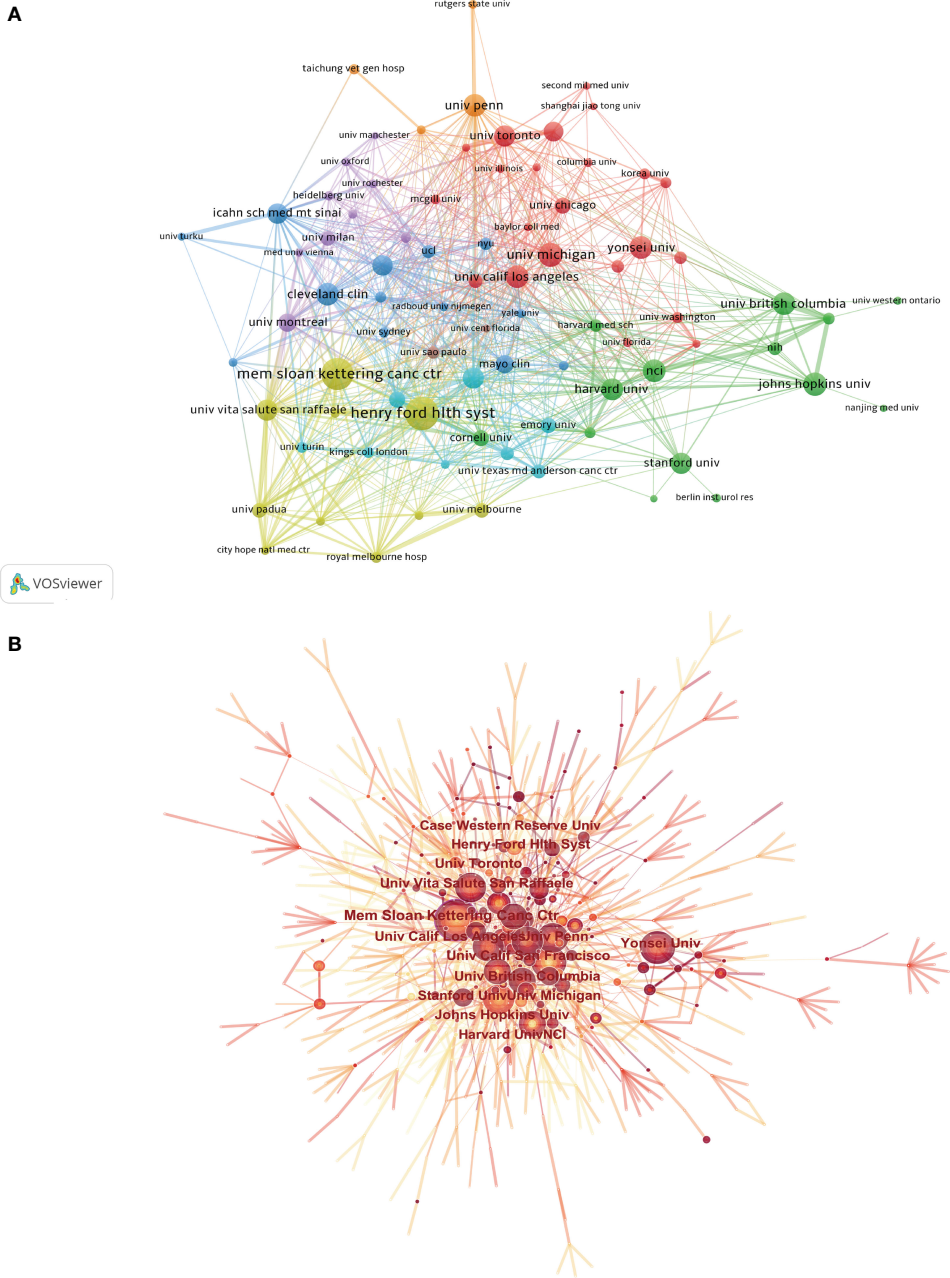
**(A)** The citation network visualization map of institutions was performed with VOSviewer. **(B)** The institutional cooperation map created with CiteSpace. The size of node represents the publication counts of an institution, and lines between nodes represent the strength of collaborations.

More than 3,000 institutions have participated in the application research of AI in PCa. **Table 2** summarizes the top 10 institutions with the highest contribution, of which the first three were Henry Ford Health System, Memorial Sloan Kettering Cancer Center, and University of Michigan, with a total of 64, 62, and 47 articles. It could be found from **Figure 4A** that there were 83 institutions with more than 15 published papers, among which the top 3 TLS were Henry Ford Health System (TLS = 101), University of Vita Salute San Raffaele (TLS = 98), and Memorial Sloan Kettering Cancer Center (TLS = 80). However, most institutions were scattered and lacked cooperation, mainly conducted in European and American institutions (**Figure 4B**). It could be found that Memorial Sloan Kettering Cancer Center was situated in a central position, and the density of the overall network was relatively low (density = 0.0023).

**Table 2:** Top 10 institutes in the publications concerning the research of AI on PCa.

Rank	Institutions	Countries/regions	Count	TLS	Total citations
1	Henry Ford Health System	USA	64	150	6,140
2	Memorial Sloan Kettering Cancer Center	USA	62	132	5,359
3	University of Michigan	USA	47	63	3,360
4	Johns Hopkins University	USA	45	65	3,396
5	Cleveland Clinic	USA	44	92	1,590
6	University of Pennsylvania	USA	44	77	1,346
7	University of British Columbia	UK	43	101	1,092
8	University of California, Los Angeles	USA	43	81	1,271
9	National Cancer Institute	USA	43	77	1,298
10	Yonsei University	South Korea	43	49	659

TLS, total link strength.

### Analysis of Authors and Co-Cited Authors

**Figure 5:**
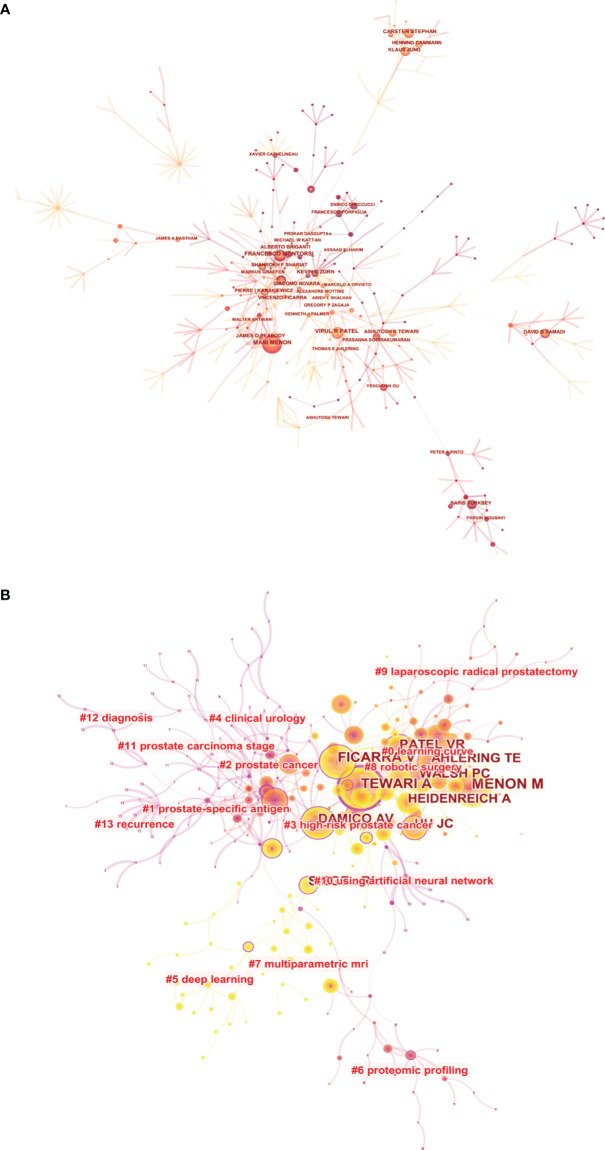
The visualization map of co-authorship **(A)** and co-citation **(B)** analyses of authors carried on CiteSpace.

A total of 12,874 authors and 39,962 co-cited authors were included in the study. **Table 3** shows the 10 most productive authors and the top 10 co-cited authors with the highest citations. Tewari Ashutosh K, Menon Mani and Patel Vipul R ranked in the top 3, with 66, 54, and 51 articles, respectively. **Figure 5A** shows that the centrality of author was lower than 0.1 and only a small amount of links could be observed in the author’s cooperation network map. The betweenness centrality (BC) is an indicator of a node’s centrality, which can reflect the importance of nodes within the networks. Generally, nodes with a BC value of more than 0.1 occupy the pivotal positions connecting a large number of nodes, and usually identified as hubs nodes displayed in purple rings (46). In co-cited author network analysis, Mani Menon, Ficarra V, and Tewari Ashutosh K were the top 3 with the highest citations. The BC of Tewari A and Kattan MW were as high as 0.4 and 0.35, respectively, indicating that the achievements of these two authors had important influence in this field. The modularity *Q* value was an index used to measure the clustering effect of the network. The larger the value is, the better the clustering result of the network. Another indicator was the silhouette value, which was used to measure the network homogeneity (47, 48). As shown in **Figure 5B**, the modular *Q* value was 0.7218, and the mean silhouette *S* value was also as high as 0.9248, indicating that the clustering effect and network homogeneity were satisfactory.

**Table 3:** The 10 most productive authors and the top 10 co-cited authors with the highest citations.

Rank	Author	Country	Count	Total citations	Co-cited author	Country	Total citations	Centrality
1	Tewari Ashutosh K	USA	66	4,137	Menon Mani	USA	737	0.09
2	Menon Mani	USA	54	6,250	Ficarra V	Italy	513	0.20
3	Patel Vipul R	USA	51	4,410	Tewari Ashutosh K	USA	498	0.40
4	Montorsi Francesco	Italy	34	3,699	Patel Vipul R	USA	356	0.17
5	Madabhushi Anant	USA	32	1,363	Walsh PC	USA	347	0.05
6	Peabody James O	USA	31	2,345	Stephan Carsten	Germany	337	0.03
7	Zorn Kevin C	Canada	30	896	Ahlering TE	USA	317	0.11
8	Stephan Carsten	Germany	30	996	D’Amico AV	USA	315	0.17
9	Karakiewicz Pierre I	Canada	29	1,443	Guillonneau B	USA	315	0.04
10	Rha Koon Ho	South Korea	26	441	Hu JC	USA	305	0.18

### Contributions of Top Journals

**Figure 6:**
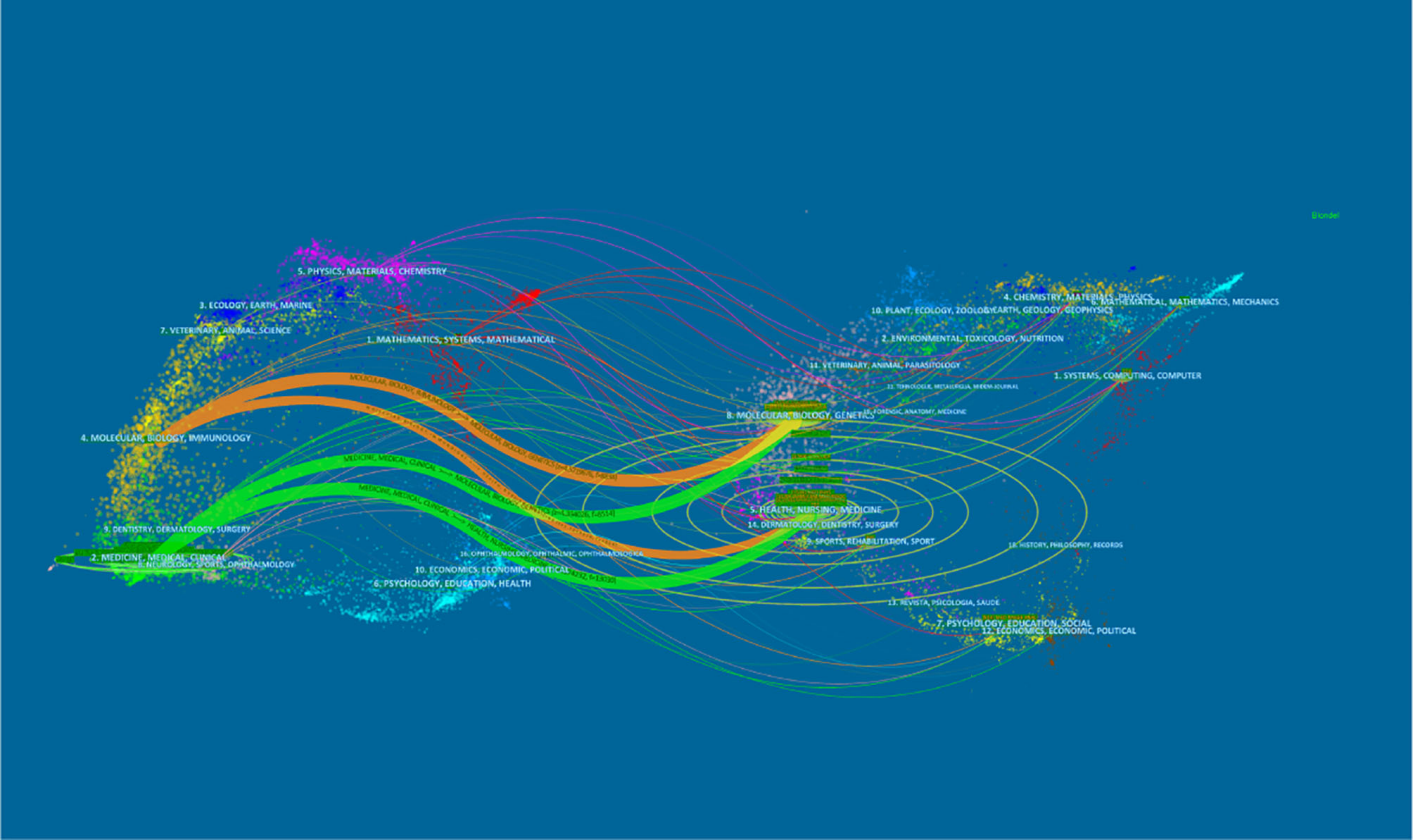
A dual-map overlap of journals on AI research in prostate cancer carried out by Citespace.

All papers were published in a total of 653 journals, including 112 journals with no less than 5 articles. According to the results of **Table 4**, the top 3 most productive journals were *BJU International* (125, 4.55%), *Journal of Urology* (106, 3.86%), and *European Urology* (100, 3.64%). Furthermore, the total citations of *European Urology* were 9,719 times, which was much higher than that of other journals. In the light of the 2020 Journal Citation Report (JCR), among the top 10 journals listed in **Table 4**, 5 journals were located in Q1. **Figure 6** is the dual map of journals, showing the relationship between citing journals and cited journals. It could be seen that there were mainly four citation paths, and the citing papers are mainly concentrated in three fields (1): Molecular, Biology and Immunology (2); Medicine, Medical, Clinical; and (3) Neurology, Sports, Ophthalmology. While the cited papers were mainly located in 3 fields (1): Molecular, Biology, Genetics (2); Health, Nursing, Medicine; and (3) Dermatology, Dentistry, Surgery.

**Table 4:** Top 10 Journals related to the research of AI on PCa.

Rank	Journal title	Countries	Count	IF (2020)	JCR (2020)	H-index	Total citations
1	BJU International	UK	125	5.588	Q1	40	5,118
2	Journal of Urology	Netherlands	106	7.45	Q1	39	5,021
3	European Urology	Netherlands	100	20.096	Q1	55	9,719
4	Urology	USA	82	2.649	Q3	32	2,734
5	World Journal of Urology	Germany	75	4.226	Q1	18	944
6	Medical Physics	UK	71	4.071	Q1	22	1,579
7	Journal of Endourology	USA	64	2.942	Q2	20	1,329
8	Urologic Oncology Seminars and Original Investigations	Netherlands	53	3.498	Q2/Q3	16	769
9	Current Opinion in Urology	USA	39	2.309	Q3	15	554
10	International Journal of Urology	UK	37	3.369	Q2	14	478

### Analysis of Top Cited References and Co-Citation References

**Figure 7:**
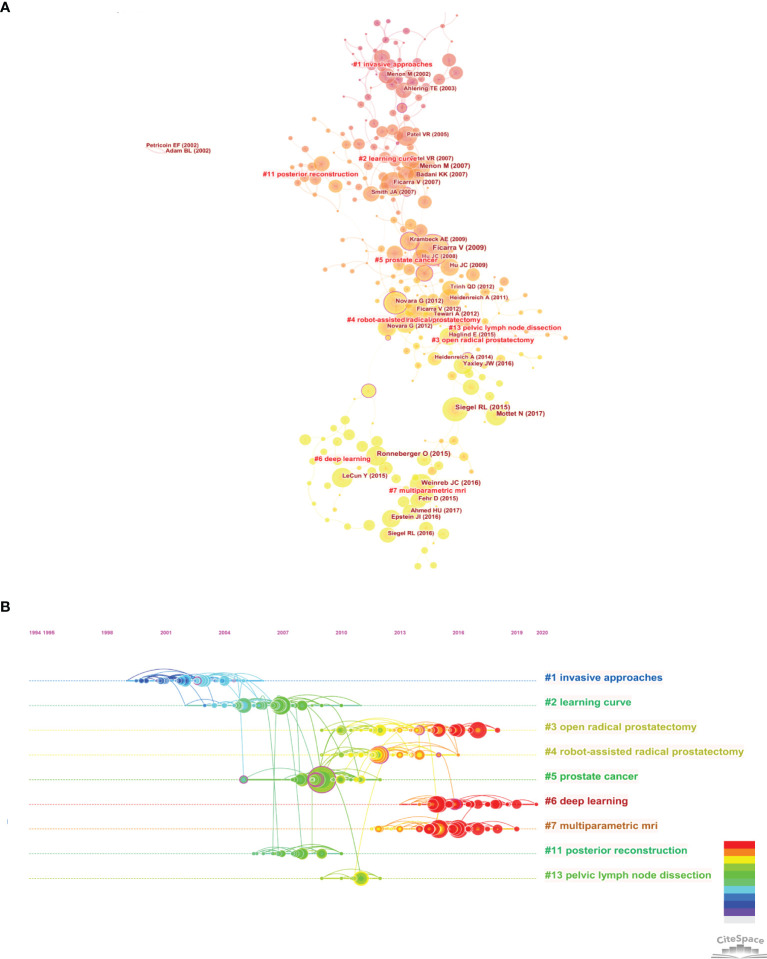
Citespace visualization map of Cluster view **(A)** and timeline view **(B)** of co-citation references. The time evolution is indicated with different colored lines, and the nodes on the lines indicate the references cited.

**Figure 8:**
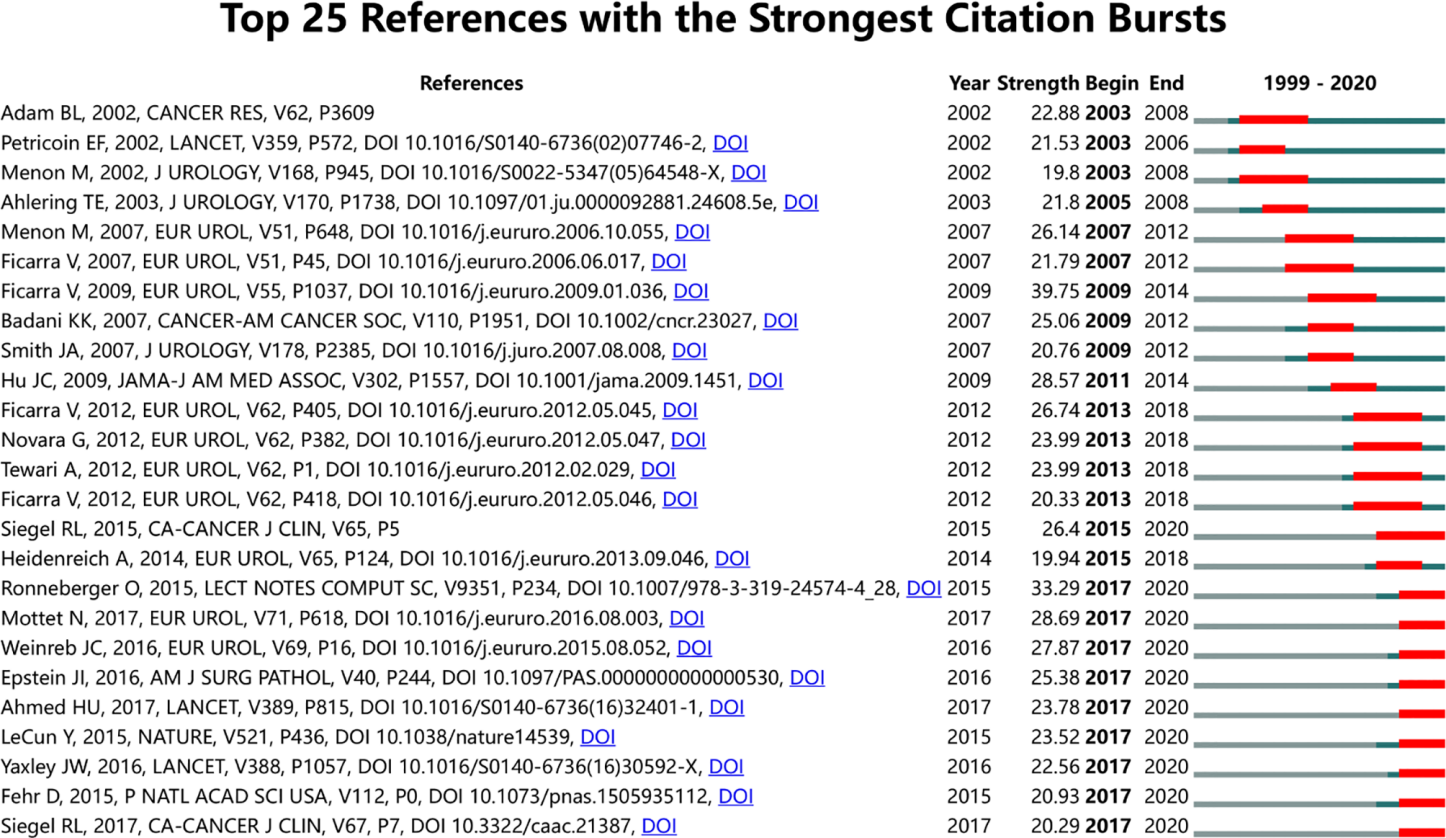
CiteSpace visualization map of top 25 references with the strongest citation bursts from 1999 to 2020.

We contained a total of 2,749 publications in this study, of which 379 documents had been cited no less than 50 times. **Supplementary Table S1** lists the top 10 papers with the highest citation, of which Tang et al. (49), with 2,288 citations, followed by Rhodes et al. (50) and Baker et al. (51) with 2,233 and 792 citations, respectively. There were altogether 62,497 cited references in our study. As shown in **Table 5**, D’Amico (52) and Ficarra (53) had the highest total citation frequency, both with 142 citations. Tewari et al. (54) ranked the second with 125 citations. **Figure 7A** visually shows the co-citation network analysis of references. From the analysis results, the Modularity *Q* was 0.8367, and the mean Silhouette S was also as high as 0.9341, showing the excellent clustering effect and good network homogeneity. **Figure 7B** also shows the timeline view of the co-citation references, reflecting the changes of research hotspots over time. According to the clustering results, it could be divided into 9 clusters. The largest cluster was “prostate cancer” (#5) (53, 55), while “invasive approaches” (#1) (56–58) was the earliest research in this field. “Deep learning” (#6) (59, 60) and “Multiparametric MRI” (#7) (61–64) were currently the latest research hotspots, suggesting that more and more researchers were paying attention to the application of deep learning and multiparametric MRI in PCa. **Figure 8** shows the top 25 references with the strongest citation bursts. The explosion of citations in this field began in 2003, and a large number of co-citation references were still being frequently cited, indicating that the application of AI in PCa was still a research hotspot in the next few years.

**Table 5:** Top 10 co-cited references concerning the research of AI on PCa.

Title	Journals	Authors	Year	Citations
Biochemical outcome after radical prostatectomy, external beam radiation therapy, or interstitial radiation therapy for clinically localized prostate cancer	Journal of the American Medical Association	D’Amico AV, etc.	1998	142
Retropubic, laparoscopic, and robot-assisted radical prostatectomy: a systematic review and cumulative analysis of comparative studies	European Urology	Vincenzo Ficarra, etc.	2009	142
A prospective comparison of radical retropubic and robot-assisted prostatectomy: experience in one institution	BJU International	A Tewari, etc.	2003	125
Classification of surgical complications: a new proposal with evaluation in a cohort of 6336 patients and results of a survey	Annals of Surgery	Daniel Dindo, etc.	2004	123
Robotically assisted laparoscopic radical prostatectomy	BJU International	J Binder, etc.	2001	119
Systematic Review and Meta-analysis of Studies Reporting Urinary Continence Recovery After Robot-Assisted Radical Prostatectomy	European Urology	Ficarra Vincenzo, etc.	2012	106
Comparative effectiveness of minimally invasive vs. open radical prostatectomy	Journal of the American Medical Association	Jim C Hu, etc.	2009	102
Laparoscopic and robot assisted radical prostatectomy: establishment of a structured program and preliminary analysis of outcomes	Journal of Urology	Mani Menon, etc.	2002	99
Successful transfer of open surgical skills to a laparoscopic environment using a robotic interface: initial experience with laparoscopic radical prostatectomy	Journal of Urology	Thomas E Ahlering, etc.	2003	97
Vattikuti Institute prostatectomy: contemporary technique and analysis of results	European Urology	Mani Menon, etc.	2007	94

### Analysis of Keyword Co-Occurrence

**Figure 9:**
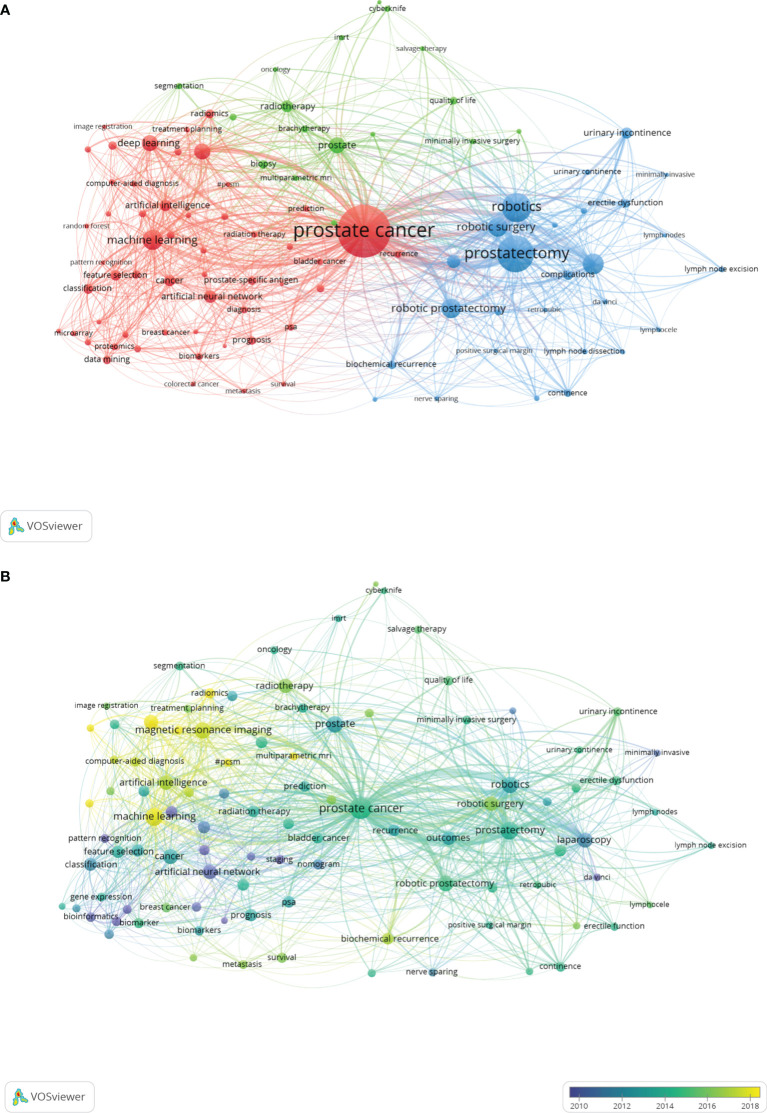
The network visualization map of the 98 keywords with a frequency of no less than 10 times generated by using VOSviewer. **(A)** All the keywords could be clustered into 3 clusters: #Cluster 1 (Diagnosis and Prediction AI-related study, red nodes), #Cluster 2 (Non-Surgery AI-related study, green nodes), and #Cluster 3 (Surgery AI-related study, blue nodes). **(B)** The overlay visualization map of keywords. The purple and blue nodes represent the keywords appearing earlier than the green and yellow nodes.

Through keyword co-occurrence analysis and burst detection, we may identify the changing trend of research topics over time, so as to better grasp the development of research hotspots. Our study included a total of 4,320 author keywords, and there were 98 keywords with a frequency of no less than 10 times. **Table 6** shows the top 20 keywords with the highest frequency. Among them, the most frequent keyword was prostate cancer with 1,269 times, followed by prostatectomy (609 times) and robotics (393 times). VOSviewer was used to generate a keywords’ network visualization map and overlay visualization map. As shown in **Figure 9A**, each color in the network visualization map represented a cluster, and all author keywords could be divided into 3 clusters. Red cluster focus on the application of AI-related technology in PCa diagnosis and prediction, for example, “diagnosis”, “deep learning”, “artificial neural network”, etc. were mainly applied to imaging analysis and disease prognosis prediction, etc. We classified it as #Cluster 1 Diagnosis and Prediction AI-related study. The green cluster mainly focused on brachytherapy or radiotherapy of prostate cancer and was defined as # Cluster 2 Non-Surgery AI-related study. The last blue cluster, with the primary keywords of “robotics”, “prostatectomy”, and “urinary incontinence”, laid particular emphasis on the surgical treatment and the postoperative conditions of PCa and could be named #Cluster 3 Surgery AI-related study. **Figure 9B** is the overlay visualization map of keywords, showing the change of keywords over time. The yellow nodes represented the emerging keywords, which indicated that these keywords may become the current research hotspots. It can be seen that “machine learning”, “deep learning”, “convolutional neural networks”, and “radiomics” were keywords that had frequently appeared in the past 3 years, suggesting that they will be the research hotspots in the future.

**Table 6:** The top 20 keywords with the highest frequency related to the research of AI on PCa.

Rank	Keywords	Frequency	TLS	Rank	Keywords	Frequency	TLS
1	Prostate cancer	1,269	5,289	11	Outcomes	78	319
2	Prostatectomy	609	2,374	12	Artificial neural network	77	356
3	Robotics	393	1549	13	Radiotherapy	69	289
4	Laparoscopy	199	807	14	Artificial intelligence	60	313
5	Machine learning	183	968	15	Cancer	57	382
6	Robotic prostatectomy	177	694	16	Urinary incontinence	57	222
7	Robotic surgery	171	658	17	Feature selection	52	266
8	Deep learning	121	555	18	Classification	45	259
9	Prostate	119	512	19	Complications	44	194
10	Magnetic resonance imaging	118	544	20	Radiomics	39	174

TLS, total link strength.

## Discussion

With the advent of the era of big data, researchers need to fully understand the development of their study field. Different from systematic review or meta-analysis, bibliometric analysis uses visual software such as VOSviewer and CiteSpace to comprehensively analyze the existing literature, so as to intuitively understand the development trend of research and predict the future research hotspots (65). This study is the first time to summarize the current application of AI in PCa through bibliometric analysis, and intuitively reveal the development trend and future research hotspots of AI in PCa by using two widely used literature measurement software tools.

In the past 22 years, AI has developed rapidly in various fields, and the application of AI in PCa has also increased exponentially (66, 67). Especially in the past 5 years, the number of publications accounted for more than half of all papers. The USA, which had the strongest productivity, published 1,342 papers, far more than other countries. In recent years, the number of papers issued by China, Germany, and other countries has gradually increased, indicating that researchers in various countries are more interested in the application of AI in PCa. It can be predicted that more countries and researchers will participate in the AI research of PCa in the future.

Advances in ML and DL have driven the rapid development of AI. The H-index (37) is an index that can be used to evaluate the level of academic output of a researcher, and it is often used to appraise the research status of researchers in a study field. Moreover, the total citation can reflect the degree of dissemination and influence of articles or journals, so as to indicate their quality and academic status. Our results showed that the USA was much higher than other countries in terms of H-index and total citation (**Table 1**). Although the number of articles published in China ranked second, the H-index was only 34, with total citations of 7,094, even lower than that in Germany (H-index=47, 10186 cited), which showed that although the number of papers in China had increased, it still lacked high-quality articles, and the main reason might be that the AI research of PCa started late in China, with an average publish year of 2017.02 (**Supplementary Figure S1**). In addition, half of the top 10 funding agencies were form the USA (**Supplementary Table S2**), which also explained why the USA could far surpass other countries in AI research. Among the top 10 most productive authors, 5 were from the USA, and the remaining authors were from Canada, Italy, Germany, and South Korea. Although China ranked second in the number of publications, there were few highly productive and cited authors. The possible reasons might be (1): the AI research of PCa started late in China, leading to a low academic influence in the world (2); the core algorithms of AI were lack of innovation, and had little cooperation with international advanced researchers; and (3) there may be certain language barriers.

Impact factor (IF), JCR category, and total citation are effective indicators to appraise journal quality. Among the top 10 journals of publications, the top 3 were *BJU International* (125, IF = 5.588, Q1), *Journal of Urology* (106, IF = 7.45, Q1), and *European Urology* (100, IF = 20.096, Q1); in addition, the total citations of *European Urology* far exceeded than that of other journals, indicating the important influence of this journal in this field. It was foreseeable that more articles on the application of AI in PCa would also be preferentially published in the above journals in the future. Moreover, *Urology, World Journal of Urology*, *Medical Physics*, and *Journal of Endourology* were also high-yield journals, and they also had the potential to publish more high-quality articles in the future to improve their academic status and impact factor.

The treatment of PCa is a global health concern (68, 69), and the application of AI in PCa will also have an important impact on the diagnosis and treatment of PCa (70). However, only 82 countries participated in the research of AI in PCa, and more than half of the countries published less than 10 papers. Except for China, the top 10 most productive countries were developed countries, showing that the research on the application of AI in PCa in developing countries was obviously lagging behind that in developed countries. Among the top 10 institutions, 8 were from the USA, which meant that the USA had the most advanced and influential research in this field. TLS is an index to measure the closeness of cooperation. From the results of co-authorship analysis, the USA was the focus in PCa research and had the closest cooperation with Italy, Germany, and Canada. The Henry Ford Health System had the closest cooperation with Memorial Sloan Kettering Cancer Center and University of Vita Salute San Raffaele, but most institutions were scattered with a density of only 0.0023, suggesting the lack of international cooperation among institutions. However, none of the top 20 institutions was from China, which indicated that Chinese institutions had little cooperation with major international research institutions. Therefore, we believed that China should actively maintain close cooperative relations with other countries, learn from the advanced technologies and research methods of other developed countries, so as to improve the influence in this field. Meanwhile, in terms of author co-authorship analysis, it could be found that the BC value of each author was lower than 0.1, indicating that even if a large number of researchers participated in the research, they were relatively scattered. As for co-cited authors, the most productive author was Tewari Ashutosh K, with a BC value of 0.4. He was mainly engaged in clinical and basal research of PCa and published a large number of articles on urinary surgery in the early phase, showing his high impact in this field (54). Kattan MW was another urologist with a high BC value of 0.35, mainly engaged in prostate cancer. He used clinical and pathological data from multicenter to model, so as to predict prostate cancer-specific mortality. It was found that poor tumor differentiation and seminal vesicle infiltration were the main determining factors of prostate cancer-specific mortality after radical prostatectomy (71). Obviously, almost all high-yielding and high co-cited authors were from the USA and European countries. However, the low-density map suggested that most researchers had less cooperation (**Figure 5A**). It is suggested that researchers from Asian countries, such as China, should strengthen cooperation with American researchers.

Co-citation analysis is often used to evaluate the relevance of articles or authors. In addition, it can be an indicator to appraise the academic influence of authors (72, 73). As shown in **Supplementary Table S1**, the most cited article in this study was Tang et al. (49), which mainly introduced a tool for data mining and analysis of TCGA and GTEX, called GEPIA. Obviously, with the advent of the big data, the processing and application of a large amount of data has become an important research method. By using the big data, we can conduct comprehensive analysis and extensive research. Through the timeline view of the co-citation references, it could be found that the earliest research focus on “invasive approaches” (#1) (56–58), and the current research hotspots were “deep learning” (#6) (59, 60) and “multiparametric MRI” (#7) (61–64), indicating that the research of early PCa mainly laid emphasis on diagnosis and treatment, and then turned to use deep learning and other technologies to achieve early diagnosis and accurate and minimally invasive treatment. The study on the frequency of keywords may reflect the development tendency of research hotspots. As shown in **Figure 9A**, we classified all keywords into three clusters, named “Diagnosis and Prediction AI-related study”, “Non-Surgery AI-related study”, and “Surgery AI-related study”. **Figure 9B** shows that the early research mainly focused on the diagnosis through PSA or other conventional screening methods and the improvement of surgical treatment methods. With the development of AI, more noninvasive examination, minimally invasive diagnosis, or treatment methods have been studied, suggesting that application of AI in PCa attracted many researchers’ attention. In other words, more and more researchers will engage in research of this field, making the diagnosis and treatment of PCa develop towards the direction of accurate diagnosis and treatment.

Big data is the basis of AI study and good data sets can train better AI models. However, the labeling and calibration of basic data requires a lot of manpower and financial resources, making data collection very difficult and precious, which may also be one of the reasons for the lack of cooperation in most studies. Therefore, the following two aspects may be the future research focus of AI. One is to develop artificial intelligence technology suitable for limited data, so that research institutions can carry out research through limited data. The other is to realize automatic labeling and calibration of data or automatic identification through unsupervised learning.

## Limitations

There are still some limitations in our study. Firstly, since it takes a certain amount time for an article to achieve a certain time of citations, the high-quality articles in recent years have not reached an ideal time of citations, which is prone to research deviation. Secondly, the exploration of the research frontier may have a time delay. Last but not least, our study only includes English literature in WoSCC, which is easy to lead to the omission of important literature in other languages.

## Conclusion

In conclusion, artificial intelligence has been widely used in prostate cancer study, especially in auxiliary diagnosis and prognosis prediction. The USA has always been in a leading position in this field and will continue to maintain the leading edge for some time in the future. However, the research cooperation intensity needs to be strengthened, especially for developing countries. They should actively maintain close cooperation with developed countries such as the USA and Italy. In addition, it should also be noted that the research focus in this field has gradually shifted from invasive diagnosis and treatment to noninvasive diagnosis and accurate minimally invasive treatment through deep learning technology.

## Data Availability Statement

The raw data supporting the conclusions of this article will be made available by the authors, without undue reservation.

## Ethics Statement

Ethical review and approval was not required for the study on human participants in accordance with the local legislation and institutional requirements. Written informed consent for participation was not required for this study in accordance with the national legislation and the institutional requirements.

## Author Contributions

TL and JK conceived the study. ZS and HW collected the data and wrote the manuscript. ZC, JH, and JP analyzed the data. TL, JK, and ZS revised and reviewed the manuscript. All authors contributed to the article and approved the submitted version.

## Funding

This study was supported by the National Key Research and Development Program of China (Grant No. 2018YFA0902803), the National Natural Science Foundation of China (Grant No. 81825016, 81961128027, 81772719, 81772728), The Key Areas Research and Development Program of Guangdong (Grant No. 2018B010109006), the Science and Technology Planning Project of Guangdong Province (Grant No. 2017B020227007), Project Supported by Guangdong Province Higher Vocational Colleges & Schools Pearl River Scholar Funded Scheme (for Tianxin Lin), and Guangdong Provincial Clinical Research Center for Urological Diseases (2020B1111170006).

## Conflict of Interest

The authors declare that the research was conducted in the absence of any commercial or financial relationships that could be construed as a potential conflict of interest.

## Publisher’s Note

All claims expressed in this article are solely those of the authors and do not necessarily represent those of their affiliated organizations, or those of the publisher, the editors and the reviewers. Any product that may be evaluated in this article, or claim that may be made by its manufacturer, is not guaranteed or endorsed by the publisher.
