# Supplementary material for: The Global Research of Artificial Intelligence on Prostate Cancer: A 22-Year Bibliometric Analysis
Source: Front Oncol. 2022 Mar 1;12:843735. doi: 10.3389/fonc.2022.843735 (PMC8921533; doi:10.3389/fonc.2022.843735)
Supplement: Supplementary file 1 [file DataSheet_1.docx]

Supplementary Material

# Supplementary Figures and Tables

## Supplementary Figures


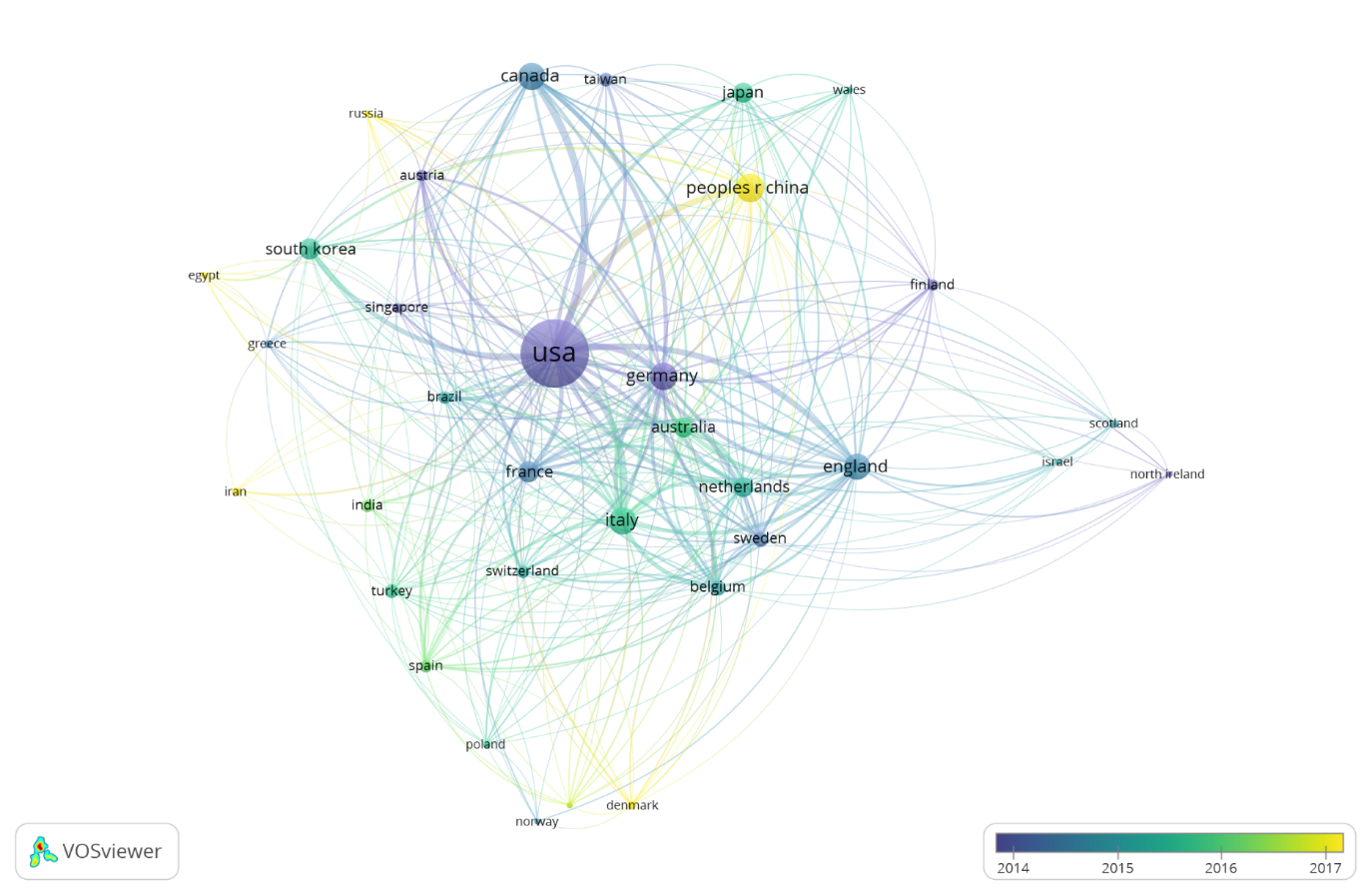


**Supplementary Figure S1.** The overlay visualization map of 34 counties/regions created with VOS viewer software.

## Supplementary Tables

**Table S1. Top 10 original articles concerning the research of AI on PCa**

| Title | Journals | Author | Year | Citations |
| --- | --- | --- | --- | --- |
| GEPIA: a web server for cancer and normal gene expression profiling and interactive analyses | Nucleic Acids Research | Tang, Zefang; Li, Chenwei; et al. | 2017 | 2828 |
| ONCOMINE: A cancer microarray database and integrated data-mining platform | Neoplasia | Rhodes, DR; Yu, JJ; et al. | 2004 | 2233 |
| Using Fourier transform IR spectroscopy to analyze biological materials | Nature Protocols | Baker, Matthew J.; Trevisan, Julio; et al. | 2014 | 792 |
| Serum protein fingerprinting coupled with a pattern-matching algorithm distinguishes prostate cancer from benign prostate hyperplasia and healthy men | Cancer Research | Adam, BL; Qu, YS; et al. | 2002 | 742 |
| Retropubic, Laparoscopic, and Robot-Assisted Radical Prostatectomy: A Systematic Review and Cumulative Analysis of Comparative Studies | European Urology | Ficarra, Vincenzo; Novara, Giacomo; et al. | 2009 | 666 |
| Systematic Review and Meta-analysis of Studies Reporting Urinary Continence Recovery After Robot-assisted Radical Prostatectomy | European Urology | Ficarra, Vincenzo; Novara, Giacomo; et al. | 2012 | 661 |
| Optimized high-throughput microRNA expression profiling provides novel biomarker assessment of clinical prostate and breast cancer biopsies | Molecular Cancer | Mattie, Michael D.; Benz, Christopher C.; et al. | 2006 | 556 |
| Mass Spectrometry as a diagnostic and a cancer biomarker discovery tool - Opportunities and potential limitations | Molecular & Cellular Proteomics | Diamandis, EP | 2004 | 501 |
| Systematic Review and Meta-analysis of Studies Reporting Potency Rates After Robot-assisted Radical Prostatectomy | European Urology | Ficarra, Vincenzo; Novara, Giacomo; et al. | 2012 | 444 |
| Systematic review: Comparative effectiveness and harms of treatments for clinically localized prostate cancer | Annals Of Internal Medicine | Wilt, Timothy J.; MacDonald, Roderick; et al. | 2008 | 410 |

**Table S2 Top 10 funding agencies for the output of the research of AI on PCa**

| Rank | Funding Agencies | Countries/regions | Count | Percentage (%) |
| --- | --- | --- | --- | --- |
| 1 | United States Department of Health Human Services | United States | 406 | 14.769 |
| 2 | National Institutes of Health | United States | 402 | 14.623 |
| 3 | Nih National Cancer Institute | United States | 302 | 10.986 |
| 4 | National Natural Science Foundation Of China | China | 114 | 4.147 |
| 5 | European Commission | European Commission | 76 | 2.765 |
| 6 | Canadian Institutes Of Health Research | Canada | 49 | 1.782 |
| 7 | Natural Sciences And Engineering Research Council Of Canada | Canada | 40 | 1.455 |
| 8 | United States Department Of Defense | United States | 37 | 1.346 |
| 9 | Nih National Institute Of Biomedical Imaging Bioengineering | United States | 36 | 1.31 |
| 10 | UK Research Innovation | United Kingdom | 35 | 1.273 |
